# Impact of environmental changes on Dermatology^☆^^☆☆^

**DOI:** 10.1016/j.abd.2020.11.004

**Published:** 2021-01-31

**Authors:** Vidal Haddad Junior, Adriana Lúcia Mendes, Carolina Chrusciak Talhari, Hélio Amante Miot

**Affiliations:** a Department of Dermatology, Faculty of Medicine, Universidade Estadual Paulista, Botucatu, SP, Brazil; b Department of Internal Medicine,Faculty of Medicine, Universidade Estadual Paulista, Botucatu, SP, Brazil; c Department of Dermatology, Universidade do Estado do Amazonas, Manaus, AM, Brazil

**Keywords:** Climatic variations, Environment, Pollution, Radiation, Sustainability, Urbanization

## Abstract

Urbanization, pollution and the modification of natural landscapes are characteristics of modern society, where the change in human relations with the environment and the impact on biodiversity are environmental determinants that affect the health-disease relationship. The skin is an organ that has a strong interface with the environment and, therefore, the prevalence patterns of dermatoses may reflect these environmental changes. In this article, aspects related to deforestation, fires, urbanization, large-scale agriculture, extensive livestock farming, pollution and climatic changes are discussed regarding their influence on the epidemiology of skin diseases. It is important that dermatologists be aware of their social responsibility in order to promote sustainable practices in their community, in addition to identifying the impacts of environmental imbalances on different dermatoses, which is essential for the prevention and treatment of these diseases.

## Introduction

Since its inception around 100-200 thousand years ago, the history of *Homo sapiens* has comprised a strong interaction with the environment, especially from migrations outside Africa, where climatic, geographical and vegetation contingencies imposed adaptive pressures that resulted in much of the diversity of the species.^1^ Without adapting to the different environmental challenges, humankind would not have occupied the entire planet.

Therefore, environmental determinants have influenced both the evolution of the species and the health of humans.^2^ For instance, the most accepted hypothesis for the differentiation of skin tones is due to the evolutionary gain resulting from the skin synthesis of vitamin D by UVB radiation and the folate photolysis by UVA radiation, selecting lighter skin colors in low-latitude regions.^3^, ^4^, ^5^ Geographic isolation, associated with genetic drift and sexual selection, were also important in defining the characteristics of major human groups.^6^ However, this genotypic variability has implicated not only in different phenotypes, but also in the development of different physiological responses, leading to disease propensities, immunologic, metabolic and therapeutic responses.^7^, ^8^, ^9^, ^10^, ^11^, ^12^, ^13^, ^14^

While the *Homo sapiens* species evolved, it intensively interacted with the environment, interfering in the health-disease relationship. Human hunter and gatherer groups (such as Pygmies and Amerindian groups) caused a discreet environmental impact, as they had a shorter life expectancy and were more exposed to environmental problems, such as accidents caused by animals, floods, infestations, zoonoses and dietary restrictions (e.g., long periods of drought).^15^

From the moment *Homo sapiens* acquired a certain domain of agriculture, fishing and domestication of animals, they began to settle into territories, establishing the first population centers. This required greater use of natural resources and modification of the local environment. Thus, there was a gain in longevity, protection against natural hazards and the possibility of territorial expansion.^16^

With the development of industrialization and changes in the means of production, there have been demographic explosions, urbanization and migratory flows to urban areas. These factors resulted in great environmental impact, causing air, soil and water pollution, in addition to the unsustainable consumption of natural resources.^17^, ^18^ From a medical point of view, the different forms of work have led to the emergence of occupational diseases and globalized transportation has disseminated infectious diseases, such as syphilis and AIDS, in addition to favoring pandemics such as the Black Plague, influenza and the disease caused by the new coronavirus (COVID-19).^19^, ^20^, ^21^, ^22^, ^23^, ^24^, ^25^

The historical condition of humankind reinforces its bilateral relationship with the environment, as well as establishes sociological and economic relations and determines specific health conditions. Considering the high degree of interaction between human skin and the external environment, Dermatology especially reflects the changes in the environment. The main impacts of environmental changes in the specialty will be discussed below.

## Environmental degradation

### Deforestation

Both urban and rural expansion modify natural landscapes, restrict native vegetation coverage, modify the geography, water and waste flow, with a direct impact on biodiversity.^26^, ^27^, ^28^, ^29^

Deforestation, pasture development, crops and underground resources exploration have been historically associated with the emergence of arboviruses, zoonoses and other infectious diseases that arise in outbreaks or endemically, depending on how the deforestation is carried out. As forests are reduced (or become unbalanced) and the reservoirs of certain diseases are extinct, humans become involved in their natural cycle.^2^, ^29^, ^30^, ^31^

Examples that emerged from this imbalance: malaria epidemics after the construction of the Panama Canal, mining and the construction of railroads in the northern region of Brazil; the yellow fever epidemic on the coast of northeastern Brazil during the 17th century sugarcane expansion and the rabies outbreak on the island of Marajó (2018), Brazil, after agricultural expansion.^32^, ^33^, ^34^, ^35^, ^36^ Likewise, the recent COVID-19 pandemic originated in an industrialized area in China (Wuhan), possibly caused by human interaction with contaminated bats as a result of restrictions in their ecosystem, which should alert humankind about the emergence of environmental issues as a priority in the sustainable development of modern society.^25^, ^37^, ^38^

**Figure 1:**
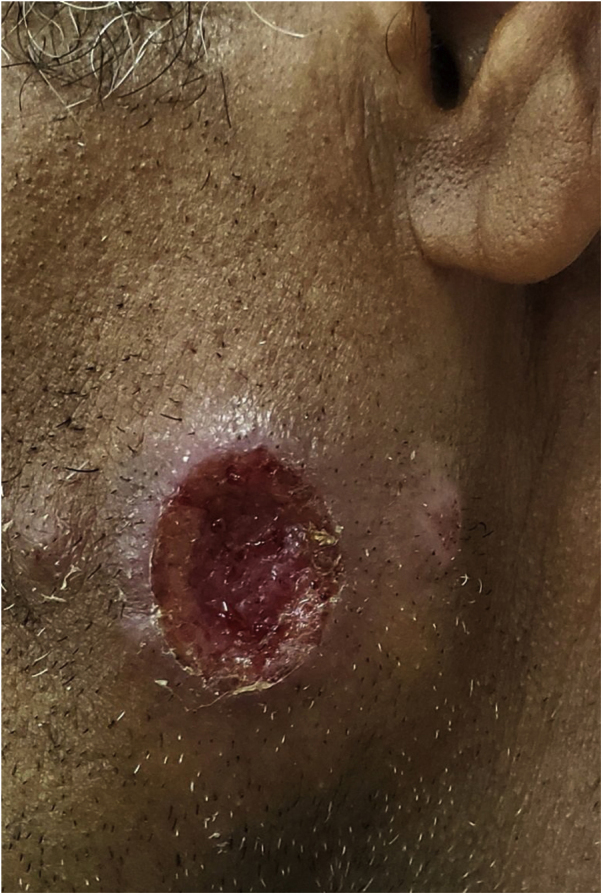
American cutaneous leishmaniasis. Facial ulcer, with an erythematous, infiltrated border and granular bottom; in a farmer from the Tietê river valley.

In Dermatology, American tegumentary leishmaniasis (ATL) is caused by protozoa of the genus *Leishmania*, which causes skin ulcers (Fig. 1) and can affect mucous membranes in later stages. It is transmitted by mosquitoes of the genus *Phlebotomus*, and has a zoonotic cycle in mammals, especially marsupials and rodents.^39^, ^40^, ^41^ In Brazil, the description of ATL was first reported during the construction of the Northwest Railway, in the beginning of the 20th century. This railroad was designed to transport the coffee production from the countryside of the Brazilian states of São Paulo and Mato Grosso. This entire area has large populations of sandflies, and the disease received the name of “Bauru ulcer” because Bauru is an important city in that region.^36^

The incidence of ATL has been increasing in the last 30 years in practically all states of Brazil, with outbreaks being described in the Southeast, Midwest, Northeast and Amazon regions. Most cases of ATL are associated with the predatory process of colonization, road construction, new population centers and expansion of agricultural activities.^42^, ^43^

Deforestation can cause the migration of infectious agents into vectors in urban areas, favoring epidemics. This has been observed with yellow fever, previously transmitted by the *Haemagogus* mosquito in wild areas, which showed phases of intense transmission in the cities by the *Aedes aegypti*.^44^ Similarly, *Aedes albopictus*, the other disseminator of viral diseases originally from Asia and Africa, was recently introduced in Brazil and is also a vector of dengue fever, zika and chikungunya.^45^, ^46^ Deforestation and the depletion of natural reservoirs is the main explanation for the occurrence of autochthonous cases of ATL in metropolitan areas, Chagas disease and (human) rabies caused by attacks of hematophagous bats in cities.^47^, ^48^, ^49^, ^50^, ^51^, ^52^, ^53^, ^54^

**Figure 2:**
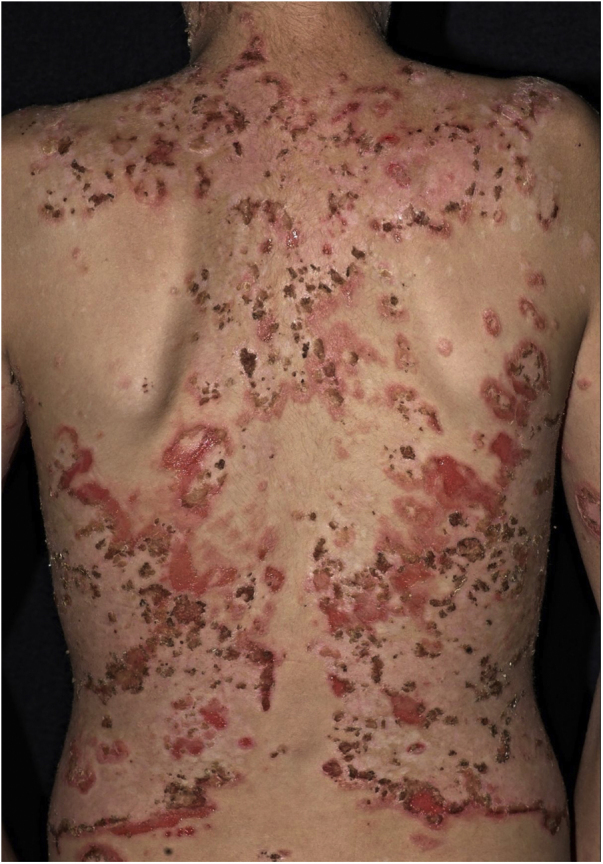
*Fogo selvagem* or endemic pemphigus foliaceus. Extensive exulcerations with hematic crusts on the back in a young resident of the Tietê river valley.

The “*fogo selvagem*” subtype of pemphigus foliaceus differs from the type described by Cazenave in that it affects younger patients (under 45 years old) and has an endemic characteristic (between longitudes 45°–60 °W and latitudes 5°–25 °S, and at altitudes between 500–800 m). Endemic pemphigus foliaceus is an autoimmune bullous dermatosis characterized by an erythematous-desquamative rash with exulcerations due to the rupture of fragile bullous lesions with a craniocaudal distribution, with photosensitivity and without mucosal involvement (Fig. 2). The pathogenesis of the disease is associated with epitope spreading, in which repeated exposure to insect bites (*Simulium nigrimanum*) would increase the production of pathogenic antibodies of the IgG4 subclass that lead to the recognition of the EC1 and EC2 domains of Desmoglein-1.^55^, ^56^ During the 20th century, there was a great increase in the incidence of the disease along areas of deforestation in the countryside of Brazil, especially in the states of São Paulo, Mato Grosso, Goiás and Minas Gerais, following large river basins.^57^

Territorial and biodiversity restrictions promoted by deforestation affect the reproduction of large predators, which demand a greater food load. Moreover, the recent description of attacks on humans by wild animals (e.g., monkeys, jaguars, anteaters, coatis) can be justified by food restrictions that affect the animals and their forced proximity to urban centers.^58^, ^59^, ^60^

The numerical restriction (or extinction) of predators may also explain the proliferation and increased incidence of accidents with scorpions, especially in the Northeast and Southeast regions of Brazil.^61^, ^62^

The occurrence of spotted fever outbreaks in the countryside of the state of São Paulo is attributed to the increase in the population of capybaras and other natural reservoirs of *Rickettsia sp*., which are protected by the prohibition of hunting. Also, with the decrease in the number of predators such as large felines, the populations of capybaras have increased exponentially and this has contributed to the spread of infected ticks (mainly *Amblyomma sp*.) spreading the disease to areas where domestic animals and the human population live.^31^, ^63^, ^64^

**Figure 3:**
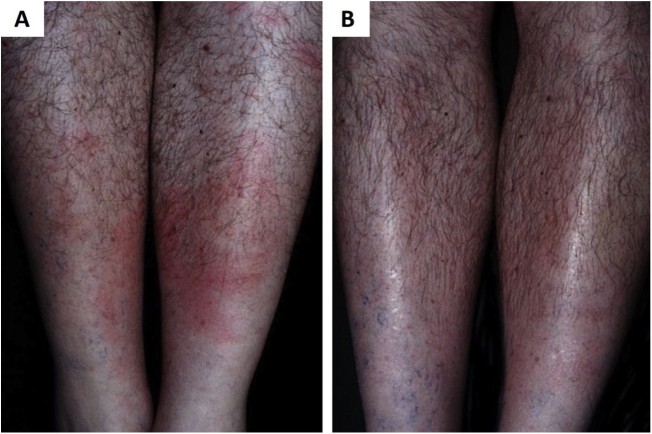
(A), *Erythema chronicum migrans* as the early manifestation of borreliosis-like illness. (B), Remission of clinical picture after treatment with doxycycline (Kindly provided by Prof. Sinésio Talhari).

As a result of the same environmental imbalance, Lyme disease in the American continent is caused mainly by the spirochete *Borrelia burgdorferi* (*sensu lato*) and transmitted by tick bites. It originally occurs among wild animals, being described in European and North American deer populations. However, it is also present in Brazil, affecting both deer and capybaras, whichspread the disease to domestic animals and humans when living close to urban centers. The disease has an early cutaneous manifestation (erythema chronicum migrans), which can trigger sclerodermiform reactions and is potentially severe (Fig. 3).^65^ The Brazilian variant of Lyme disease (borreliosis-like illness, or Baggio-Yoshinari syndrome) needs further studies, especially since its incidence seems to be underestimated considering the number of clinical manifestations that may not be exactly the same as classic Lyme disease (European or North American).^66^, ^67^, ^68^, ^69^, ^70^

Brazil has the largest freshwater network in the world. Due to this fact, the main national energy matrix was developed based on the construction of hydroelectric power plants, especially starting from the 1950s, leading to the flooding of more than 34,000 km^2^.^71^ Large water dams, however, promote profound and damaging changes to the geography and the riverside ecosystem. The influence of the changes in the local microclimate (temperature and rainfall) will be discussed later. Changes in the aquatic fauna, both by reducing river flows and by the non planned introduction of fish species, are environmental determinants of health problems.^72^, ^73^, ^74^

**Figure 4:**
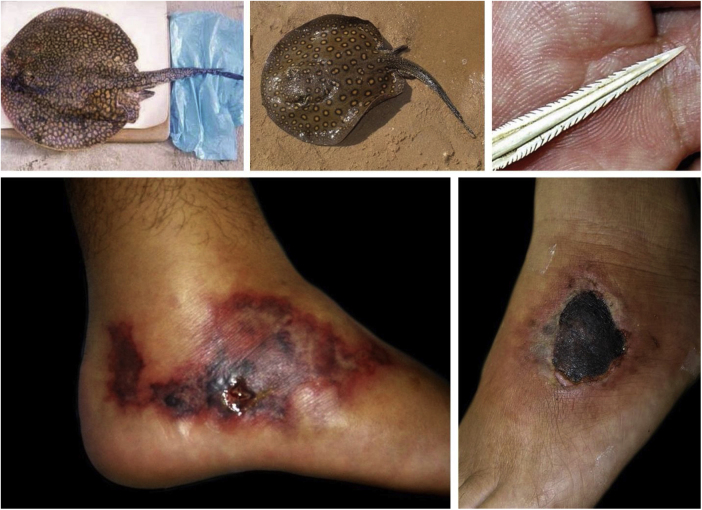
Freshwater stingrays (*Potamotrygon sp*.) associated with severe accidents with bathers and fishermen at the Paraná River basin. Detail of the serrated stinger. Skin lesions due to stingray accidents: lower-limb ulcers on an extensive livedoid base (< 72 h) that develops into necrosis and eschar (> 7 days).

**Figure 5:**
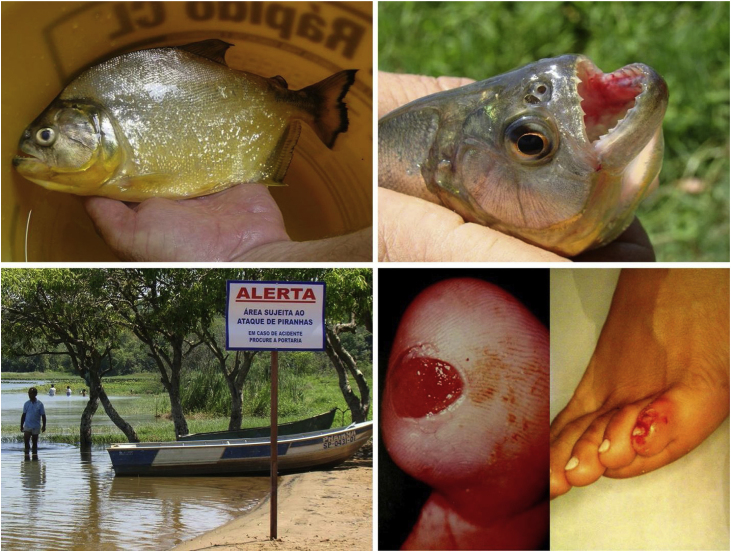
River resort (municipality of Adolfo-SP, 21°09'56"S, 49°43'03"W) at a reservoir area of the Tietê River. Accidents due to piranha attacks on bathers: punched-out ulcers in the foot. Details of the adult animal and the triangular teeth (*Serrasalmus maculatus*).

Freshwater stingrays, for instance, are native animals from the North and Midwest regions of Brazil. After the construction of rainwater dams, natural barriers were reduced. This favored the mobilization of fish downstream the Paraná river, precipitating severe accidents involving fishermen and bathers in the Southeastern region, including the state of São Paulo and the Tietê river (Fig. 4). ^75^ Similarly, piranhas have settled in dammed areas and have caused a series of accidents among bathers in the summer season (Fig. 5).^76^, ^77^

Finally, deforestation has also changed the regional rainfall cycle, where the impact on biodiversity extends beyond the deforested area. In the beginning of the 20th century, the city of São Paulo was known as the “land of drizzle” due to the intense rainfall resulting from the surrounding dense Atlantic forest and its rich hydrography. Currently, it is the largest megalopolis in Latin America, and the extensive urban “heat island” has substantially modified the region's microclimate. The progressive deforestation of the Amazon region is also noticeable with the reduction of the pluviometric index in the Northern region of Brazil that has occurred in the last 50 years.^78^

A process of deforestation of small areas used by indigenous and *quilombola* populations is the burning of vegetation (*coivaras*), which consists in a controlled and restricted use of fire. The fire is limited by firebreaks, which are deforested areas to interrupt the continuity of combustion, causing minimal environmental damage. However, the recent fires in extensive areas of the Brazilian Pantanal and the Amazon rainforest (as in Australia and Argentina) are attributed both to spontaneous combustion during drought periods and to the deforestation practice by residents, mainly for the expansion of pastures and agriculture.^79^

The reduction of rainfall favors the spread of fire outbreaks, which in addition to the massive air pollution and damage to biodiversity, promote the rapid migration of wild animals and disease vectors fleeing their burning habitat. In this context, the chances of accidents with animals and the transmission of zoonoses in urban areas near the burned regions increase.

### Large-scale agriculture

The production of food for the planet’s current population demand depends on the improvement of production techniques and this includes the mechanization of crops, genetic engineering and the use of pesticides.

Since the 1950s, pesticides have been gradually included in Brazilian agriculture. However, the training of farmers for their use and management has not accompanied this trend, causing damage to human health and the environment. The lack of individual protection when handling the pesticides favors skin and respiratory toxicity.^80^, ^81^

**Figure 6:**
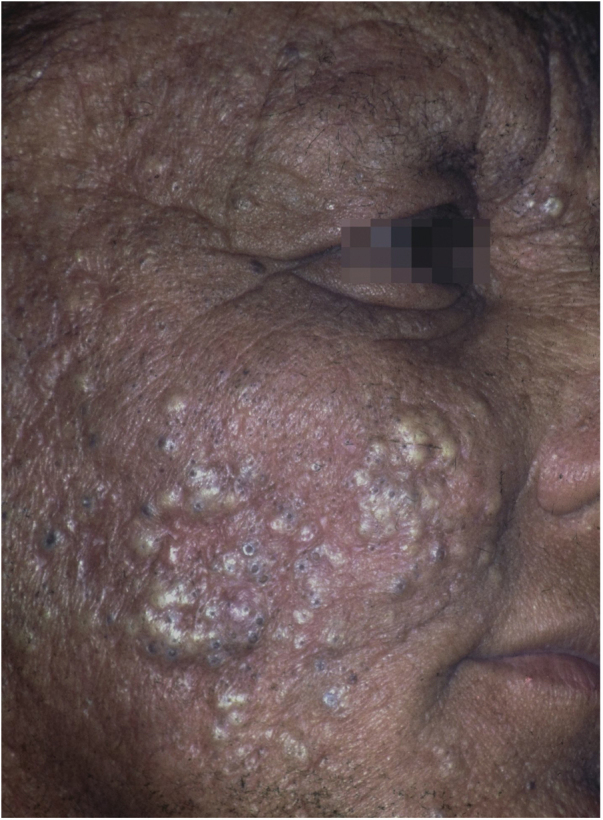
Chloracne. Farmer from the Tietê river valley with extensive papulopustular eruption with comedones. He reports unprotected handling of pesticides containing hexachlorobenzene (banned from use in Brazil in the 1980s). Three other family members were affected.

Occupational or industrial exposure to chlorinated hydrocarbons (dinitrophenol, pentachlorophenol) leads to chloracne-like acneiform rashes (Fig. 6).^19^ The handling of organophosphates, carbamates, pyrethroids and dipyridyls leads to the development of allergic and irritant contact dermatitis.^80^ Exposure to pesticides has also been identified as a risk factor for some types of skin cancer, including squamous cell carcinoma and melanoma. Arsenic is believed to be the main carcinogen involved in this process.^82^, ^83^

The mechanization of agriculture has greatly reduced the direct contact of farmers with the land and vegetation, which, in addition to requiring fewer professionals to perform the same activity, has also required greater professional qualification in the field. In the context of Dermatology, besides reducing occupational accidents and those caused by poisonous animals, the mechanization has also promoted a reduction in cases of deep mycoses in rural areas.^84^ The incidence of paracoccidioidomycosis has been steadily declining in the last 30 years, also because there is the interference of air humidity, water reservoirs and atmospheric pressure on the viability of fungi in the soil. States in the Amazon region (e.g. Rondônia), still maintain the most significant indicators due to a more most recent development of the agricultural activity.^85^, ^86^, ^87^

Genetic modifications of seeds (transgenic plants), the cultivation of non-native species, large-scale agriculture and extensive livestock farming demand deforestation and promote an important reduction in biodiversity, favoring the emergence of health problems.

### Urbanization

**Figure 7:**
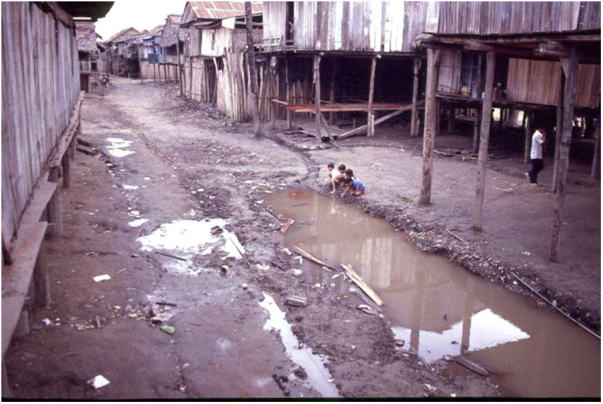
Non planned urbanization process in an indigenous village in the upper Solimões region, state of Amazonas (Kindly provided by Prof. Sinésio Talhari).

The Brazilian demographic transition of the last century was characterized by rural-urban population migration, industrialization and the modification of the age pyramid. Few municipalities, however, showed a planned and sustainable development, resulting in problems related to housing, access to health, drinking water and basic sanitation (Fig. 7).^88^, ^89^ This situation constitutes a major challenge for public health policies because it depends on the knowledge and modification of its social determinants, instead of just promoting diagnoses and offering medications.^90^

Large urban agglomerations as well as the need for means of mass transportation (e.g., trains, buses) constitute challenges for the control of diseases with respiratory transmission such as tuberculosis and COVID-19; their progression in large cities is more evident than in municipalities with low population density.^91^, ^92^

**Figure 8:**
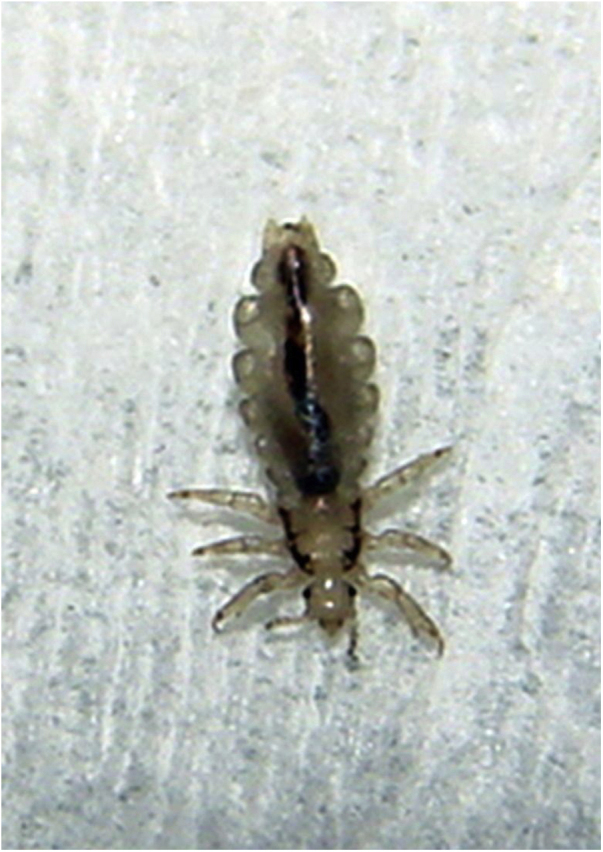
*Pediculus humanus capitis*. Causal agent of pediculosis of the scalp, endemic in large urban agglomerations.

In Dermatology, ectoparasitoses are mainly influenced by urban agglomerations and poor sanitary conditions. The prevalence of scabies in the slums in the Brazilian Northeast region affects up to 8.8% of residents, while pediculosis can affect 43.4%.^93^ As a complicating factor, the indiscriminate use of pyrethroids to treat pediculosis of the scalp allowed the emergence of resistant strains of *Pediculus humanus* var. *capitis*, resulting in greater difficulty in infestation control and in restricting the epidemic (Fig. 8).^94^, ^95^

Sexually-Transmitted Infections (STIs), especially syphilis, Human Papillomavirus (HPV) anogenital warts, Human Immunodeficiency Virus (HIV), gonorrhea, non-gonococcal urethritis (e.g., *Chlamydia sp*.) and genital herpes have shown an increased incidence in the last two decades in several countries.^96^, ^97^ The economic development associated with urbanization favors the increase of prostitution and more sexual intercourse, maximizing the risk of STI transmission.^2^, ^98^, ^99^ The formation of new communities of workers for the construction of hydroelectric power plants, mining parks or planned cities (e.g., Brasilia, Teresina and Palmas) have historically accompanied an increase in firearm violence and STIs.^100^, ^101^, ^102^, ^103^

**Figure 9:**
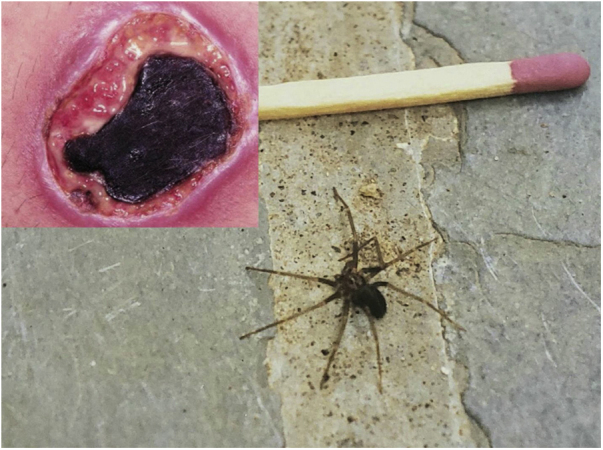
Skin ulcer with necrotic eschar formation derived from an accident caused by a spider of the genus *Loxosceles* (>96 h). Example of an adult brown spider (*Loxosceles sp*.).

Some animals previously found only in rural areas have adapted to cities due to the lack of predators and to obtain food supplies from produced by humans such as the *Loxosceles* spider, which cause severe accidents with ulcer formation and kidney damage in its victims (Fig. 9).^104^ Certain cities such as Curitiba, in the southern region of Brazil, record thousands of accidents annually.^105^ Another example is the proliferation of pigeons from Europe and North Africa (*Columba livia*) in urban areas, increasing the risk of systemic mycoses such as cryptococcosis and infestations such as gamasoidosis, which have been recorded in several parts of the country.^106^, ^107^, ^108^, ^109^

The non planned urbanization of municipalities promotes important changes in the natural landscape, with an impact that goes beyond biodiversity and affects the local microclimate. The systematic drainage of water courses associated with extensive waterproofing of the soil and reduction of the vegetation cover, modify both the thermal stabilization capacity of water bodies and the terrestrial albedo (solar energy reflection coefficient). These elements promote a significant increase in temperature, amplified by the reduction of air circulation resulting from the successive construction of skyscrapers and by the production of heat from human activity (e.g., automobile traffic). The formation of these heat islands resultant from urbanization can change the temperature in the center of an urban area by more than 6 °C in relation to the adjacent rural area, with an important decrease in humidity and a reduction in the dispersion of air pollutants, which can favor several risks to human health.^110^, ^111^, ^112^, ^113^, ^114^, ^115^

**Figure 10:**
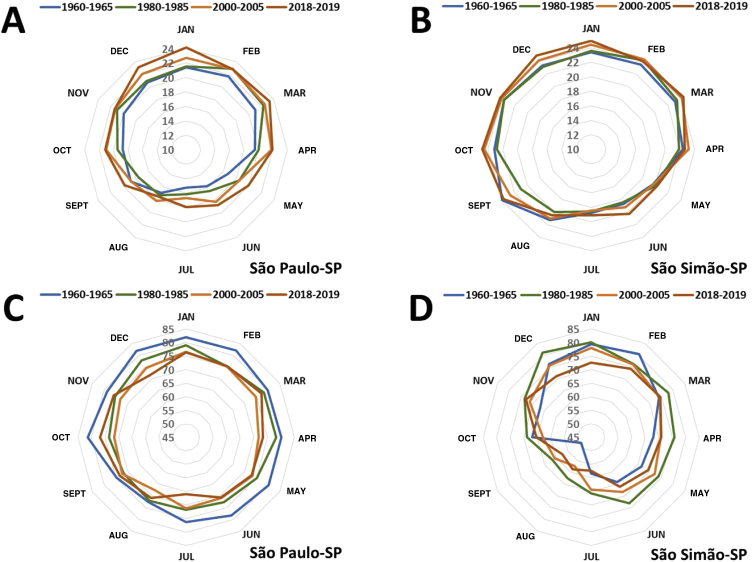
Temporal series (1960 to 2019) of monthly temperature averages (A and B) and relative humidity (C and D) in the municipalities of São Paulo-SP and São Simão-SP. (Source: INMET).

Fig. 10 shows the effect the heat island inflicted on air temperature and humidity in the last 70 years in the metropolitan area of the municipality of São Paulo (Latitude: 23°32'56”S, Longitude: 46°38'20”W; altitude: 745 m), compared to the small rural municipality of São Simão (Latitude: 21°28'41”S, Longitude: 47°33'3”W; altitude: 663 m). There has been a consistent and progressive increase in the average temperature (+ 3.2 °C) and a reduction in the relative humidity of the air (-10%) in the metropolitan area, while the temperature and humidity naturally oscillated during the temporal series in the rural municipality.

The modification of the terrestrial albedo by construction of buildings and the paving of soil also promotes greater reflection of ultraviolet radiation, which potentially worsens photoinduced dermatoses, such as melasma, rosacea, lupus erythematosus and the skin field cancerization.^84^, ^116^

Large cities (especially megalopolises) create vast islands of heat and air pollution, which are primarily associated with diseases linked to hypersensitivity, such as asthma, conjunctivitis and atopic dermatitis.^117^, ^118^, ^119^ The specific effects of air pollution on the skin will be discussed below.

Heat islands also interfere with the activity and reproduction of insects and arachnids. In Curitiba, the number of accidents by brown spiders showed a seasonal behavior, especially when the surface temperature exceeded 30 °C.^120^

The effect of heat on cultural rites linked to clothing (suits, synthetic clothes, closed shoes) and the manner of working (e.g., poorly ventilated production lines), promotes sweating and increased sebum production, which favor bacterial and fungal infections.

**Figure 11:**
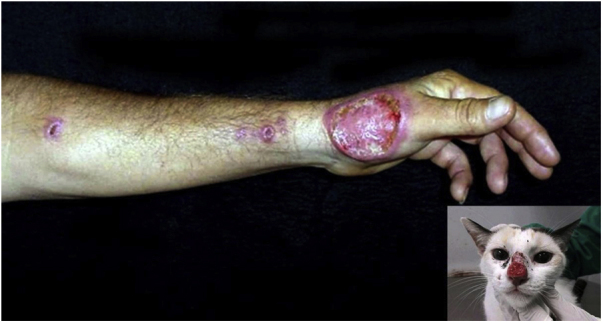
Cutaneous-lymphatic sporotrichosis, ulcer on the left hand dorsum, accompanied by ascending nodular lymphangitis in a young adult male who “adopted” an infected street animal. Picture detail showing the cat with an ulcer on the nose, a characteristic representation of feline sporotrichosis.

Urbanization, when associated with the lack of control of proliferation of stray domestic animals such as dogs and cats, in parallel with the lack of predators in the urban environment, also favors the emergence of zoonoses. Sporotrichosis is a subcutaneous mycosis caused by the fungus *Sporothrix schenckii*, more frequently found in countries with tropical or subtropical climates. In the skin, it manifests mainly as ulcers and ascending nodular lymphangitis (Fig. 11).^121^

In Brazil, sporotrichosis of occupational origin, caused by trauma with vegetables (“gardener’s disease”) had its incidence reduced to the detriment of contamination by animals, which is on the rise among young adults, especially due to scratches or bites by contaminated cats, the main agents involved in this zoonotic chain.^122^ Felines have a high fungal load due to the habit of scratching trees, traveling long distances and engaging in fights, which favors contamination. The access of non-neutered cats to the streets, the abandonment or sacrifice of sick animals by the guardian, inadequate disposal of carcasses, associated with the lack of integration between the Epidemiological Surveillance and the Zoonosis Control Center of the municipalities are acts of negligence that contribute to the dynamic of disease transmission. Zoonotic sporotrichosis has been described in several states in Brazil and has become endemic in the Southeastern Region of the country, especially in Rio de Janeiro, in the last 20 years.^123^

Similarly, as a result of great urban mobility (such as international travel), lack of predators and emergence of resistance to pyrethroids, usually employed in residential pest control, bedbug (*Cimex lectularius*) bite epidemics have been described in several urban centers around the world.^124^, ^125^ Cimidiasis clinically manifests as pruritic edematous papules, especially on the extremities, which can assume a linear aspect (the bug’s “breakfast, lunch and dinner”), typical of fleas and bedbugs.^126^, ^127^ An additional concern regarding skin hypersensitivity reactions is the possibility that bedbugs can be vectors of other infectious diseases, evidence that is not yet a consensus among researchers.^128^, ^129^, ^130^

### Pollution

One of the most damaging effects of modernity is the compromising of our environment (soil, air and water) with residues from human production. Environmental changes do not occur in isolation in the community. Deforestation, reduction of biodiversity, urbanization and pollution of the environment are usually shown to be interrelated as marks of modern human activity. Water contamination, noise pollution and waste disposal are of the utmost importance in public health.^131^, ^132^, ^133^, ^134^, ^135^

From the dermatological viewpoint, the skin is affected by particulate air pollution and volatile gases, especially nitrogen dioxide (NO_2_), sulfur dioxide (SO_2_), ozone (O_3_), and carbon dioxide (CO_2_). The main mediators of air pollution in the skin are the Aryl Hydrocarbon receptors (AhR) present in all skin structures, which are activated by aromatic hydrocarbons such as dioxins, widely present in vehicular smoke.^136^, ^137^

Air pollution contributes to extrinsic skin aging, and mostly derives from the burning of fossil fuels and industrial activity in urban centers. Skin that is submitted to intense air pollution has a barrier deficit, with less squalene production, in addition to oxidative consumption of tocopherol and the formation of lentigos and wrinkles.^136^, ^138^ In the dermis, particulate pollution can induce inflammatory phenotypes in fibroblasts, with increased metalloprotein (MMP-1, MMP-3) synthesis, reduced collagen (COL1A1, COL1A2), and elastin synthesis.^139^

The specific damage from air pollution depends on the type of pollutant, the skin integrity and the intensity of exposure.^140^ Aromatic hydrocarbons promote different epithelial stimuli; in addition to the abovementioned ones, there is the formation of epoxy and diols which bind directly to DNA, promoting epigenetic changes in cell growth, potentiating the development of neoplasms, especially if there is joint stimulation with ultraviolet radiation.^141^, ^142^, ^143^

In addition to aging and carcinogenesis, air pollution favors the development of inflammatory dermatoses, such as eczema and acne. Aromatic hydrocarbons, especially dioxins, are known to induce acne mediated by AhR activation, acting on sebocytes, endothelium and epidermis.^143^, ^144^, ^145^ Individuals who traveled to areas with high air pollution levels reported an inflammatory acne outbreak. In Beijing, demand for acne care has been correlated with higher indicators of air pollution.^144^

The incidence of atopic dermatitis is influenced by low humidity, temperature and pollutants. Particulate pollution consists of numerous salts, heavy metals and aromatic hydrocarbons, which penetrate the skin through hair follicles and acrosyringea complex. Sweat increases the transepidermal penetration, promoting oxidative damage and inducing an inflammatory response in the dermis and epidermis, which culminates in damage to the barrier function.^110^, ^137^ In a four-year Chinese temporal series, the demand for eczema care was associated with daily air pollution indicators.^146^ Pollution is one of the factors responsible for the increased incidence of atopic dermatitis in Europe, which occurs more significantly in urban centers than in rural communities.^131^, ^140^

Forest fires, whether accidental or controlled fires aiming deforestation for agriculture, promote, in addition to deforestation, a reduction in biodiversity, reduction in soil water content and launch a large amount of gases and particles into the air. This shows that changes in the environment are not only measurable due to their direct effects, but also on the entire ecosystem as well.

In addition to the ozone that pollutes the atmosphere, promoting oxidative damage to the skin with a reduction in microflora and consumption of tocopherol, ozone also naturally forms in the stratosphere, around 20 to 30 km above sea level depending on the reaction of solar ultraviolet radiation with atmospheric oxygen.^147^ It plays a major role in completely blocking UVC radiation emitted by the sun (extremely mutagenic) and approximately 90% of UVB radiation. This equilibrium between ozone synthesis and degradation in the stratosphere ensures that a tolerable amount of mutagenic radiation reaches the planet surface.^148^

The ozone layer that circumscribes the Earth is not homogeneous, and has thinner areas, especially at the poles. There is an intense discussion about the cyclical variation in the configuration of the ozone layer and its degradation by air pollutants. Atmospheric emissions of halogenated compounds such as chlorofluorocarbon (CFC), halon, hydrochlorofluorocarbon (HFC), methyl bromide, carbon tetrachloride (CTC), methyl chloroform and hydrobromofluorocarbons (HBFC) have been associated with the reduction of the ozone layer in the atmosphere.^148^ The use of these compounds in soft drinks, propellants and foams has been reduced since the 1990s worldwide, with the expectation of complete interruption of use in the next 30 years.

It is estimated that a 1% reduction in the ozone layer promotes an increase of 2% in the incidence of UVB and, consequently, an increase of 2% in the incidence of skin cancer.^149^ In fact, in inhabited regions with lower concentrations of stratospheric ozone, higher rates of cutaneous and mucous neoplasms (basal cell carcinoma, squamous cell carcinoma and melanoma) are recorded in humans and animals, demanding greater stringency in photoprotection strategies.^150^, ^151^, ^152^, ^153^, ^154^

### Climatic changes

Climate can be defined as the set of atmospheric changes in a certain region of the planet such as temperature, precipitation and winds, with patterns that tend to be repeated in a certain period of time (e.g., it is hot and rainy in January, in the city of Rio de Janeiro). Conventionally, the climatic behavior is evaluated in 30-year periods, and the main characteristic of the climate is its natural variability according to the years, with a complex interaction between the factors that determine it. It is important to differentiate “climate” from “meteorological weather”, which considers the state of the local atmosphere at a given time (e.g., today has been the hottest day of the year in the city of São Paulo).

The variation in the temperature of the oceans (the El Niño and La Niña phenomena and the decadal oscillation of the Pacific Ocean), solar activity (solar cycles), orbital trajectory (more circular or more elliptical), Earth’s axis inclination, volcanic activity and gravitational changes (lunar cycles) are the main global climate modifiers.^155^, ^156^, ^157^, ^158^, ^159^, ^160^ Its main determinants in a given region comprise the latitude, altitude, continentality (distance from the coast), cloud density, ocean temperatures, sea currents, vegetation/urbanization (albedo), water masses and geography.^111^, ^115^, ^161^, ^162^ This justifies the immense variability of climatic characteristics around the planet.

During the evoltion of humankind several global climate changes were recorded, such as small ice ages in Europe between 540 and 550 and between 1350 and 1850 due to less solar activity, changes in the Earth’s orbit and greater volcanic activity.^163^, ^164^, ^165^ There have also been significant warming periods, such as the Roman Warm Period (between 250 BC to 400 C.E.), and the medieval period (between 800 and 1200 C.E.) detected in the northern hemisphere. All of these changes resulted in social, economic and health impacts on the population.

There is currently an intense discussion with geopolitical repercussions on whether the focal environmental changes promoted by human activity can influence global climate, to the detriment of the evident abovementioned loco-regional changes and the natural variability of the planet’s climate cycles. However, this specific discussion goes beyond the scope of this article.

There is a clear seasonality in the incidence of dermatological diseases: psoriasis shows lower prevalence or severity in summer, due to ultraviolet radiation. However, there is a higher incidence of staphylococcal infections, actinic keratoses and accidents by venomous animals due to the type of leisure activities practiced during this time of the year. In contrast, there are more respiratory infections during the winter, when immunological imbalance favors leprosy reactions.^166^, ^167^, ^168^ It is therefore expected that climatic changes may interfere with the incidence of dermatoses.

Humidity and the increase in temperature constitute factors known to influence the rate of reproduction and activity of mosquitoes, the main vectors of infectious diseases.^169^, ^170^ A temporal series showed that the incidence of ATL in the Amazon region was strongly influenced by warm temperatures and changes in rainfall patterns caused by El Niño.^171^

In another series, in Peru, with 3,294 cases (2004–2007), the incidence of viral warts, actinic keratosis, rosacea and eczema was influenced by the climatic phenomena of the Pacific (El Niño / La Niña).^172^ These studies show that changes in regional microclimates promoted by deforestation, flooding/damming, agglomerations and urban heat islands are potential environmental determinants in the incidence of dermatoses.

In conclusion, environmental changes have an impact on human health/disease association, included in an ecological context. Currently, urbanization, large-scale agriculture, pollution of the environment and deforestation are the environmental determinants that should have the greatest impact on the incidence of dermatoses.^173^ Dermatologists should be aware of their social responsibility in order to promote sustainable practices in their community, in addition to identifying the environmental imbalances that favor each dermatosis, which is crucial for the prevention and treatment of these diseases.

## Financial support

None declared.

## Authors' contributions

Vidal Haddad Junior: Study concept, writing and approval of the final manuscript.

Adriana Lúcia Mendes: Study concept, writing and approval of the final manuscript.

Carolina Chrusciak Talhari: Study concept, writing and approval of the final manuscript.

Hélio Amante Miot: Study concept, writing and approval of the final manuscript.

## Conflicts of interest

None declared.
